# Donor T cells for CAR T cell therapy

**DOI:** 10.1186/s40364-022-00359-3

**Published:** 2022-04-01

**Authors:** Tiffany C. Y. Tang, Ning Xu, Robert Nordon, Michelle Haber, Kenneth Micklethwaite, Alla Dolnikov

**Affiliations:** 1 Graduate School of Biomedical Engineering, Faculty of Engineering, UNSW Sydney, Sydney, NSW Australia; 2 Children’s Cancer Institute, Lowy Cancer Research Center, UNSW Sydney, Sydney, NSW Australia; 3 School of Women’s and Children’s Health, Faculty of Medicine, UNSW Sydney, Sydney, NSW Australia; 4 Kids Cancer Center, Sydney Children’s Hospital, Sydney, NSW Australia; 5 Blood Transplant and Cell Therapies Program, Department of Hematology, Westmead Hospital, Sydney, NSW Australia; 6 Blood Transplant and Cell Therapies Laboratory, NSW Health Pathology, ICPMR Westmead, Sydney, NSW Australia; 7 Westmead Institute for Medical Research, Sydney, NSW Australia; 8 Sydney Medical School, The University of Sydney, Sydney, NSW Australia

**Keywords:** Donor CAR T cells, Genome editing, CRISPR-Cas9, TALENs, GVHD

## Abstract

Adoptive cell therapy using patient-derived chimeric receptor antigen (CAR) T cells redirected against tumor cells has shown remarkable success in treating hematologic cancers. However, wider accessibility of cellular therapies for all patients is needed. Manufacture of patient-derived CAR T cells is limited by prolonged lymphopenia in heavily pre-treated patients and risk of contamination with tumor cells when isolating T cells from patient blood rich in malignant blasts. Donor T cells provide a good source of immune cells for adoptive immunotherapy and can be used to generate universal off-the-shelf CAR T cells that are readily available for administration into patients as required. Genome editing tools such as TALENs and CRISPR-Cas9 and non-gene editing methods such as short hairpin RNA and blockade of protein expression are currently used to enhance CAR T cell safety and efficacy by abrogating non-specific toxicity in the form of graft versus host disease (GVHD) and preventing CAR T cell rejection by the host.

## Background

Chimeric antigen receptor (CAR) T cells have shown remarkable efficacy in treating B cell malignancies such as B cell acute lymphoblastic leukemia (B-ALL), B cell non-Hodgkin lymphoma (NHL), mantle cell lymphoma (MCL), follicular lymphoma (FL) and multiple myeloma (MM), although more improvements are needed for treating chronic lymphocytic leukemia (CLL) [1–19]. However, currently approved clinical treatments are expensive and complicated to manufacture, delaying patient access to treatments. This has prompted a need to investigate options for widening accessibility for all patients using donor sources to manufacture CAR T cells. Healthy donor peripheral blood (PB) is currently used to generate CAR T cells in preclinical and early phase clinical studies, but in addition to traditional uses in transplantation, umbilical cord blood (UCB) presents an untapped source of healthy donor T cells for adoptive immunotherapy and can be used to create a bank of readily available off-the-shelf CAR T cells. Both gene editing and non-gene editing approaches can be used to enhance CAR T cell function and eliminate alloreactivity from allogeneic donor-derived CAR T cells, making them safe for administration into patients and reducing their rejection by the host immune system.

## CAR T cell therapy

CAR T cell immunotherapy offers potentially curative treatments for refractory leukemia and lymphomas. In the clinic, T cells isolated from patient PB can be genetically engineered to express CARs that specifically target tumor antigens [5, 6, 10, 20–25]. After ex vivo amplification to numbers suitable for adoptive cell therapy, these autologous CAR T cells are infused back into the patient, where they become living drugs that detect and kill tumor cells, even in advanced stages of disease [5, 6, 10, 20–25]. Approximately 80% of patients with relapsed or refractory B-ALL (r/r B-ALL) and 40–60% of patients with relapsed or refractory diffuse large B cell lymphoma (DLBCL) showed complete responses after anti-CD19 CAR (CAR19) T cell treatment [5, 6, 10, 20–25]. As a result, the FDA has recently approved 3 autologous anti-CD19 CAR T cell therapies: tisagenlecleucel (Kymriah, Novartis) for treating pediatric and adolescent (age 25 or under) B-ALL and adult DLBCL; axicabtagene ciloleucel (Yescarta, Gilead) for treating DLBCL, NHL, and FL; and brexucabtagene autoleucel (Tecartus, Gilead) for treating adult relapsed or refractory (r/r) MCL [1–6, 8–19, 26–28]. The FDA has also approved idecabtagene vicleucel (Abecma, Bristol Myers Squibb), an autologous anti-BCMA CAR T cell therapy, for treating adult r/r MM [7, 29].

**Fig. 1:**
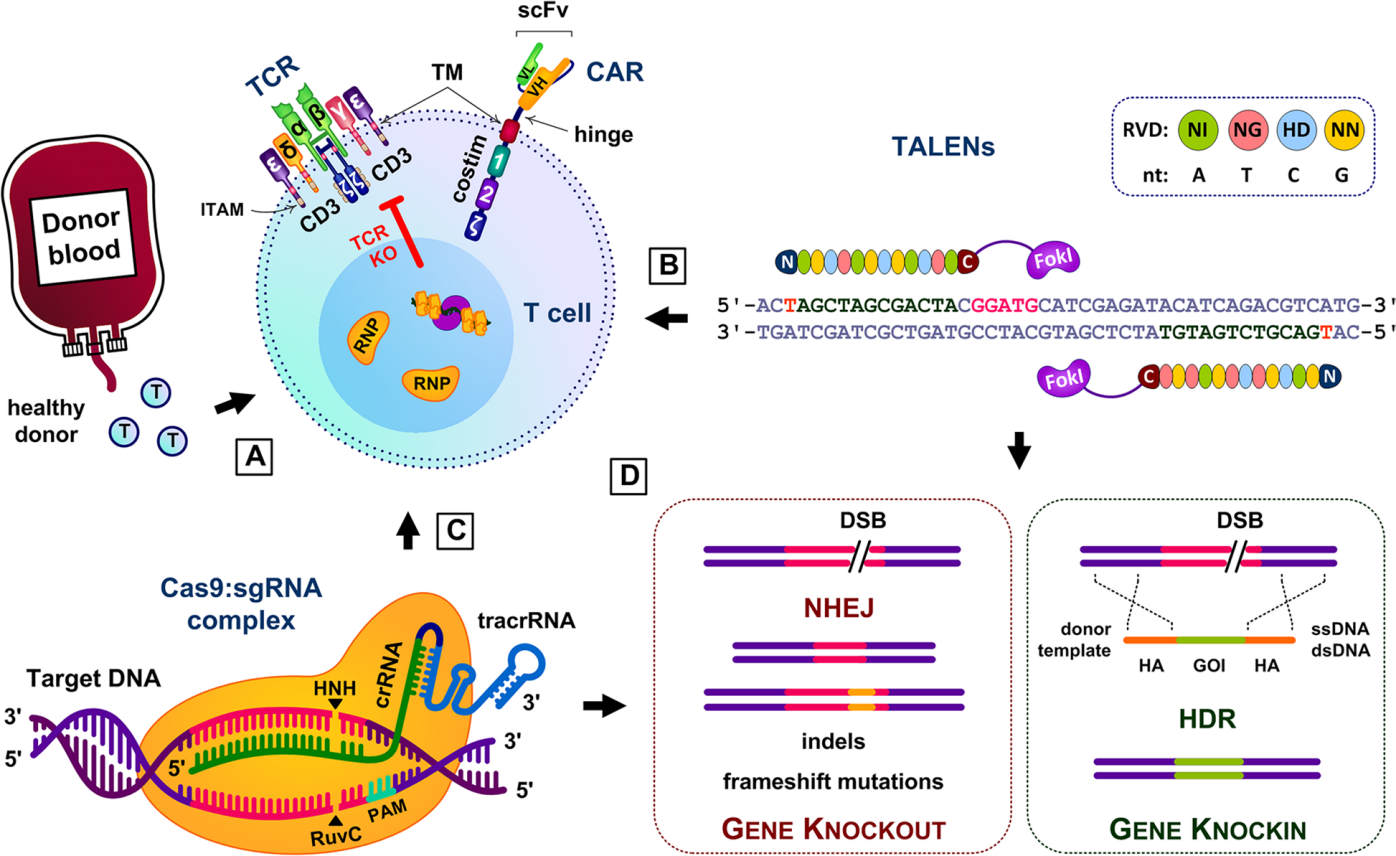
Creating universal CAR T cells with genome editing. **A** Healthy donor T cells isolated from PB or UCB are genetically modified to express CAR. VL and VH chains are linked by a flexible peptide to form the scFv that recognizes tumor antigens. The hinge connects the scFv to the TM that anchors the receptor to the T cell’s membrane. TCR-derived CD3ζ and one or more co-stimulatory signaling domains activate CAR T cells. **B** To avoid alloreactivity, TCR-KO CAR T cells can be generated using genome editing techniques such as paired TALENs, composed of TALEs fused to *Fok*I endonucleases for targeted DNA cleavage. **C** TCR KO can also be achieved using CRISPR-Cas9. Cas9 endonucleases and sgRNA form RNP complexes that cleave DNA at HNH and RuvC nuclease active sites. **D** DSBs from DNA cleavage are repaired via NHEJ or HDR mechanisms.

CARs are fusion proteins typically combining extracellular monoclonal antibody-derived targeting fragments with intracellular signaling domains that activate T cells (Fig. 1A). Variable light (VL) and heavy (VH) chains are linked by a flexible peptide to form a single chain variable fragment (scFv) that recognizes and binds to tumor antigens [30, 31]. The scFv is connected via a hinge or spacer to the transmembrane domain (TM) that anchors the CAR to the T cell membrane. The hinge provides flexibility for the scFv to reach tumor antigens and, along with the TM, provides stability for CAR expression. The hinge and TM are typically extracellular domains like CD8α (Kymriah) or CD28 (Yescarta) that avoid Fcγ receptor (FcγR) binding activity, in order to circumvent off-target effects and improve CAR T cell engraftment, persistence, and antitumor efficacy. Beneath the TM are intracellular co-stimulatory and T cell receptor (TCR) derived CD3ζ signaling domains that are crucial for CAR T cell activation, proliferation, differentiation, survival, and persistence. First-generation CARs consist only of CD3ζ while second and third-generation CARs include additionally 1 and 2 co-stimulatory domains respectively [30, 31]. Commonly used co-stimulatory domains include 4-1BB (Kymriah), CD28 (Yescarta), ICOS, OX40, or CD27.

Clinical CAR T cell therapeutics such as Kymriah and Yescarta are commonly manufactured using lentiviral or gamma-retroviral vectors, respectively, to transfer CAR genes to T cells [32]. Protocols are safe and optimized, but are complicated by the time and expense needed to ensure that all viral vectors used are replication-deficient; thus production of these CAR T cells is mostly centralized [32]. Automated cell processing platforms such as the CliniMACS Prodigy® (Miltenyi Biotec) and the Cocoon® Platform (Lonza) can expedite and scale-up CAR T cell manufacture, but issues of manufacturing standardization and product characterization remain [33].

Ex vivo CAR T cell manufacture is challenged by problems in generating high enough numbers for infusion while maintaining viability and high antitumor efficacy with minimal exhaustion. The cells generated are also a mix of helper (CD4^+^) and cytotoxic (CD8^+^) T cells. Preclinical studies by Pfeiffer et al. and Agarwal et al. present an alternative solution by using lentiviral vectors to selectively generate CD8^+^ or CD4^+^ CAR T cells in vivo [34, 35]. Interestingly, Agarwal et al. showed that CD4^+^ CD19-targeting CAR T cells have higher antitumor efficacy at high tumor burden than CD8^+^ CAR T cells alone [34]. Some mice in Pfeiffer et al.’s proof-of-concept study displayed symptoms indicative of cytokine release syndrome (CRS), an acute inflammatory syndrome that can cause multi-organ dysfunction in some patients as a serious side effect of CAR T cell therapy [35]. An advantage of in vivo CAR T generation is the slower manifestation of CRS, since CAR T cell levels increase homeostatically and thus more gradually than that of adoptive cell therapy, where patients are infused with high numbers of ex vivo activated CAR T cells [35]. Another recent study by Nawaz et al. demonstrated that CAR T cells can also be generated in vivo using adeno-associated virus (AAV) vectors in a similar mouse model with promising results showing high efficacy against T cell leukemia, which is immensely difficult to treat using current CAR T cell therapies [36]. Successful clinical translation of in vivo generation of CAR T cells can help significantly in solving the challenges of ex vivo CAR T cell manufacture, widen patient access to immunotherapies, and improve clinical outcomes.

Compared with viral vectors, non-viral transposon-based gene delivery systems are simpler, cheaper, have no infectious potential, and enable CAR T cells to be produced by hospitals for wider patient access to CAR T therapies [37]. Transposon-based methods have similar DNA integration profiles to viral vectors, but can carry larger cargoes (up to 200 kb) and simultaneously deliver multiple transgenes, which will be useful as future generations of CAR T cells become more sophisticated [37]. CAR T cells engineered using transposon-based systems to target CD19^+^ leukemia and lymphomas have demonstrated strong efficacy in preclinical mouse models and early phase clinical trials in USA, Australia, and China [37–40]. Recent Phase I clinical trial (ACTRN12617001579381) data showed 9 out of 10 patients achieving complete remission after treatment with donor-derived piggyBac transposon-based CAR19T cells against r/r B-ALL or aggressive lymphoma post-HSCT; however, 2 patients developed malignant CAR19T cell-derived lymphoma that resulted in the death of 1 patient while the other was successfully treated [41, 42]. Thus the risk of oncogenicity of piggyBac transposon-based CAR T manufacture presents a safety challenge that must be overcome for further clinical applications.

## Healthy donor peripheral blood or umbilical cord blood as sources of allogeneic CAR T cells

Despite the aforementioned successes, autologous CAR T cell production is often not feasible for heavily pre-treated patients, as shown by interim analyses of the Phase II ELIANA trial (NCT02435849) for pediatric r/r ALL that revealed CAR T cell manufacture failed in approximately 8% of patients [43, 44]. In the setting of B cell malignancies, prolonged lymphopenia in chemotherapy-treated patients can limit the generation of potent autologous CAR T cells, with CD8^+^ T cells taking at least 3–6 months to recover post-chemotherapy and CD4^+^ T cells taking even longer [45–48]. Additionally, T cells derived from chemotherapy-treated patients are often more differentiated compared with those derived from healthy blood, and demonstrate lower ex vivo and in vivo proliferative capacity and rapid exhaustion following antigen-specific stimulation. Thus harvesting lymphocytes from patients earlier in their treatment may provide a better source of lymphocytes for CAR T cell manufacture. Alternatively, using healthy donor peripheral blood may provide high numbers of cells with stronger proliferative capacity. Other donor cell sources, such as UCB, may also be considered for CAR T cell development. The advantages and disadvantages of autologous and allogeneic CAR T cell therapies are summarized in Table 1 [49–52], with comparison of clinical trial data in Table 2.

**Table 1:** Advantages and disadvantages of autologous and allogeneic CAR T cell therapies [49–52]

Autologous CAR T cells	Allogeneic CAR T cells
Manufacture is complex and expensive with variability in starting material (patient T cells) and resulting CAR T cell product. Limited T cell quality and quantity (autologous PBMCs from leukapheresis product) with risk of manufacture failure for heavily pre-treated patients (lymphopenia).	Standardized manufacture with high quality starting material (healthy donor T cells) and high quality CAR T cell product. Multiple T cell sources from many healthy donors (PB or UCB).
Low scalability (1 product per patient) with increased time to treatment and production costs due to manufacture and quality control specific for individual patient.	High scalability (1 product for many patients) with “off-the-shelf” bank of CAR T cell products, readily available (decreased time to treatment). Reduced production costs with manufacture and quality control applicable to many patients.
Risk of contamination with malignant cells in patient blood.	Minimal risks of malignant cell contamination since T cells are sourced from healthy donor blood.
Limited optimization of T cell phenotype and function with limited editable cancer targets, promising applications in B cell malignancies but limited applications in T cell malignancies.	High optimization of T cell phenotype and function to improve CAR T efficacy with multiple editable cancer targets, e.g. promising applications in both B and T cell malignancies.
Limited potency of CAR T cell product due to chemotherapy-treated patient T cells being more differentiated with lower proliferative capacity and rapid exhaustion. Increased in vivo persistence compared with allogeneic CAR T cells due to lack of immune rejection from the host.	Potent CAR T cell product from healthy donor T cells, but with decreased in vivo persistence due to higher immunogenecity (from the host against infused CAR T cells).
CRS or CRES toxicities. Low immunogenicity and minimal risk of alloreactivity or immune rejection affecting clinical outcomes.	CRS or CRES toxicities. Risk of alloreactivity factors (e.g. GVHD, immune rejection) affecting clinical outcomes.

**Table 2:** Comparison of efficacy, persistence, and toxicity associated with autologous and allogeneic CAR T cell therapies in selected recent clinical trials

CAR T cell therapy	Target antigen	Malignancy	CAR expression technology	Gene editing strategy to mitigate negative alloreactivity factors	Efficacy	CAR T cell expansion and persistence	Toxicities	Phase	References
**SCRI-CAR19v1** Autologous	CD19	r/r B-ALL, r/r NHL	Lentiviral transduction of CAR19 construct into activated CD4 and CD8 enriched T cells derived from autologous PBMCs in leukapheresis product.	Not applicable	**Phase I:** Overall 40/45 (89%) MRD^–^ CR remission rate; 93% MRD^–^ CR in CAR T-treated patients and 100% in patients who also had fludarabine and cyclophosphamide lymphodepletion. **Phase II:** Anti-tumor response in 5/6 patients at 3 weeks, with CR by Week 9 in 2 DLBCL patients but not sustained despite CAR T persistence; 1 CR patient developed new CD19^+^ site at 6 months but CR at 3 weeks post second CAR T infusion; 1 PR patient developed CD19^–^ at 9 weeks.	**Phase I:** Functional CAR T persistence until CD19^–^ relapse 8 months post-infusion. **Phase II:** All patients showed CAR T cell expansion in PB, BM, and CSF with ongoing persistence at 14 days to 9 months.	**Phase I:** Reversible severe CRS and/or reversible severe neurotoxicity in 23% patients; no patient deaths from toxicity. **Phase II:** CRS: Grade I (*n* = 3), Grade II CRS (*n* = 1), Grade III CRS (*n* = 1). Neurotoxicity: Grade I (*n* = 1), Grade 2 (*n* = 1), no severe neurotoxicity.	**Phase I:** 45 children and young adults **Phase II:** 8 patients (4–18 yrs): r/r DLBCL (*n* = 4), Burkitt's lymphoma (*n* = 2), gray zone B cell lymphoma (*n* = 1), primary mediastinal B cell lymphoma (*n* = 1)	**NCT02028455** Gardner et al. (2017) [22] Rivers et al (2018) [53]
**P-BCMA-101** Autologous	BCMA	r/r MM	Non-viral piggyBac transposon delivers anti-BCMA CAR (fused with the less immunogenic Centyrin protein to CD3ζ/4-1BB) with a safety switch to autologous T cells harvested from leukapheresis, while retaining high %T_SCM_	Not applicable	57% ORR for 34 patients treated with single P-BCMA-101 during initial dose escalation; 4 patients treated with cyclic P-BCMA-101, rituximab, lenalidomide, or single P-BCMA-101 at lowest dose showed 100% ORR with ongoing responses and minimal CRS.	Patients treated with rituximab or lenalidomide pre- and post-lymphodepletion showed gradual T_SCM_ expansion (peak at 2–3 weeks and detectable for up to 1.5 years) and increased T cell robustness.	CRS in 17% patients: Grade III in 1 patient, neurotoxicity in 1 patient, 3 patients treated with tocilizumab, no patients admitted to ICU admission or needed safety switch activation. No patient deaths or off-target toxicities. 79% Grade III neutropenia, 30% thrombocytopenia, 30% anemia.	**Phase I/II (PRIME):** 43 patients (67% male, 33% female, median age 60 yrs)	**NCT03288493** Costello et al. (2019–2021) [54–56]
**UCART19** Allogeneic	CD19	r/r B-ALL	Recombinant lentiviral transduction of CAR19 (4-1BB) with CD20 target mimotope for rituximab (safety switch) into healthy donor T cells.	*TRAC* and *CD52* KO using mRNA encoding TALENs to disrupt TCRαβ expression to limit GVHD, while making the CAR T cells resistant to anti-CD52 monoclonal antibody lymphodepletion.	14/21 (67%) patients with CR or CR with incomplete hematological recovery 28 days post-infusion; patients (*n* = 4) not given alemtuzumab showed no UCART19 expansion or anti-leukemic activity; median duration of response 4.1 months with 10/14 (71%) responders proceeding to allo-SCT; progression-free survival 27% at 6 months; overall survival 55%.	Rapid UCART19 expansion in blood with peak at Day 14 and reduction by Day 28 with persistence in some patients; 15/17 (88%) patients treated with fludarabine, cyclophosphamide, and alemtuzumab showed UCART19 expansion; no UCART19 expansion in patients treated with only fludarabine and cyclophosphamide but showed indications of earlier host lymphocyte recovery; larger AUC for first 28 days post-UCART19 in responders than non-responders; UCART19 persisted past Day 42 in 3 patients with 1 patient showing detectable UCART19 at Day 120.	CRS in 19 patients (91%); Grade III–IV CRS in 3 patients (14%); neurotoxicity in 8 patients (38%); Grade I acute skin GVHD in 2 patients (10%); Grade IV prolonged cytopenia in 6 patients (32%); 1 death from neutropenic sepsis with concurrent CRS; 1 death from pulmonary hemorrhage with persistent cytopenia.	**Phase I (PALL):** 7 children **Phase I (CALM):** 14 adults	**NCT02808442** **NCT02746952** Benjamin et al. (2020) [57]
**CTX110** Allogeneic	CD19	r/r DLBCL, LBCL, Grade 3B FL	CAR19 transgene construct inserted into *TRAC* locus of donor T cells using multiplex CRISPR-Cas9 editing (no lentivirus or retrovirus).	CRISPR-Cas9 mediated *TRAC* KO to disrupt TCR expression to mitigate GVHD, and *B2M* KO to disrupt β-microglobulin and remove MHC-I expression to limit rejection and enhance CAR T cell persistence.	Single CTX110 dose at level 2+ (intent-to-treat) achieved 58% ORR and 38% CR rate in LBCL; CR rate 21% at 6 months; 4/9 patients achieved CR at Day 28 and stayed in CR at 6 months (remaining 5 patients not reached 6-month evaluation yet); longest response rate at 18 months.	CTX110 expansion or persistence kinetics not yet reported.	No GVHD; only Grade I–II CRS and resolved with standard management; CTX110 re-dose did not increase CRS frequency or severity; Grade III+ ICANS in 1 patient with concurrent HHV-6 encephalitis; no ICANS at dose levels 3–4; 1 patient with pseudomonal sepsis (resolved in 4 days).	**Phase I (CARBON):** recruiting; treated 29 adult patients so far	**NCT04035434** McGuirk et al. (2021) [58] CRISPR Therapeutics (2021) [59]

AUC: area under the curve; BM: bone marrow; CR: complete response; CRS: cytokine release syndrome; CSF: cerebrospinal fluid; GVHD: graft versus host disease; ICANS: immune effector cell-associated neurotoxicity syndrome; KO: knock out; ORR: overall response rate; PB: peripheral blood; PR: partial response; T_SCM_: T stem cell memory

Manufacture of CAR T cells for T cell malignancies faces unique challenges due to the similarities between normal and malignant T cells. CAR T cells that target antigens common between normal and neoplastic T cells may kill both tumor T cells and CAR T cells [60, 61]. This fratricide, or mutual killing of CAR T cells, may prevent the generation and expansion of CAR T cells during the manufacture process. However, the targeting of malignant T cells without killing normal or CAR T cells can be achieved by using CAR T cells that have been genetically edited ex vivo to prevent expression of the T cell target [60, 61].

Contamination with tumor cells is an additional concern in refractory leukemia patients, since T cells are isolated from PB that may contain malignant blasts. This has resulted in at least one case of accidental expression of CAR in leukemic B cells leading to epitope masking and relapse, but is especially problematic in T cell malignancies where selection steps in CAR T manufacture using CD3, CD4 or CD8 are also likely to enrich leukemic cells and cause manufacture failure [32, 62]. Moreover, the peripheral blood of patients suffering from T cell acute lymphoblastic leukemia (T-ALL) or T cell lymphoma (TCL) often contain neoplastic T cells that may inadvertently be harvested and transduced with CAR, which can competitively bind to the target antigens on malignant T cells. The challenges of isolating healthy T cells from the phenotypically identical neoplastic T cells can be avoided by transfecting NK cells or healthy donor T cells [60, 63–67].

CAR T cells can potentially be manufactured from the peripheral blood mononuclear cells (PBMCs) of healthy donors that can be stored and validated before use, and infused into multiple patients immediately as needed. However, donor CAR T cell-mediated graft versus host disease (GVHD) and recipient-mediated rejection of CAR T cells needs to be eliminated to make allogeneic CAR T cells safe and effective. CAR T cells derived from a matched sibling donor have been safely used to treat patients who relapsed after allogeneic hematopoietic stem cell transplantation (allo-HSCT), but outside of the setting of past transplant, the identification of a suitable sibling followed by CAR T cell manufacture is even more logistically challenging than autologous CAR T cells and no less expensive [68–70].

UCB can be a new source of healthy donor T cells for developing effective immunotherapies. Compared with those derived from adult blood, UCB-derived T cells have more naïve phenotype, higher proliferative capacity, delayed exhaustion following antigen-specific stimulation, lower immunogenicity and reduced risk of inducing GVHD [71–73]. Up-regulation of T cell exhaustion markers decreases CAR T cell persistence, limiting the efficacy of CAR T cell therapy and increasing the risk of relapse [74]. We have demonstrated that CAR19 T cells up-regulated PD-1 and TIM-3 exhaustion markers in co-cultures with CD19^+^ leukemia cells and in leukemia patient-derived xenograft (PDX) mouse models [74]. UCB T cells generally express lower levels of T cell inhibitory receptors compared to those of adult PB [72]. Importantly, UCB T cells also mount more effective antitumor responses via faster tumor infiltration with CCR7^+^ CD8^+^ T cells and faster induction of cytotoxic CD8^+^ T cells and CD4^+^ Th1 cells in the tumor microenvironment [72]. All these factors make the readily available UCB potentially more advantageous than other T cell sources.

Studies have shown that CAR T cells can be efficiently produced from UCB. UCB-derived T cells co-expressing endogenous TCR against common viruses that affect patients post-SCT, for example, cytomegalovirus (CMV), Epstein-Barr virus (EBV), and adenovirus (AdV), were genetically modified with CD19-targeting CAR [75]. Allogeneic UCB-derived CAR19 T cells were infused in patients with B cell malignancies after SCT in a recently completed clinical trial (NCT01362452), showing the potential of UCB T cells for cancer immunotherapy and especially in combination with SCT [76].

## Methods to reduce CAR T cell alloreactivity

Adoptive transfer of donor-derived CAR T cells can be compromised by potential risks of alloreactivity due to the diverse TCR repertoire expressed by mature T cells [77, 78]. The TCR on adoptively transferred donor T cells may recognize recipient tissues as foreign and induce a cytotoxic immune reaction known as GVHD [77, 78]. GVHD is caused by expanding alloreactive donor T cells that infiltrate and destroy host tissues such as those in the skin, liver, and gut [77, 78]. In myeloablative conditioned recipients of haploidentical hematopoietic stem cell transplantation (haplo-HSCT) with post-transplant cyclophosphamide (PTCy) as GVHD prophylaxis, grades II–IV and III–IV acute GVHD were higher after ≤ 5/8 and 6–8/8 HLA-matched UCB HSCT, while chronic GVHD was comparable between donor sources [79]. Similarly, grade III–IV acute GVHD was higher in recipients of haploidentical relative donor HSCT than in recipients of matched unrelated donor (MUD) HSCT [80]. Many variables such as conditioning, T cell depletion and GVHD prophylaxis can affect the rates of GVHD with HLA-haploidentical or UCB transplants, but most importantly, all allogeneic T cell sources carry a risk of GVHD without some form of manipulation.

Despite toxicity issues such as hypotension and fever, HLA-matched donor-derived CAR T therapy for patients with relapsed B cell malignancies [68, 81–85] post allo-HSCT have lower rates of GVHD compared to those expected with unmanipulated donor lymphocyte infusions [41, 82, 86, 87]. This does not seem to be the case in donor-derived CAR T cells for T cell malignancies, where grade I-II acute GVHD was seen in 60% of recipients of CD7-specific T cells [88]. These results are from small, early-phase studies with variation in manufacturing protocols and CAR design, but they highlight the need for further technical advances to eliminate the risk of GVHD.

GVHD is not the only alloreactivity factor affecting clinical outcomes. Recent murine studies demonstrating potent graft-versus-lymphoma (GVL) activity with reduced xenogeneic GVHD of donor-derived CAR19 T cells in allo-HSCT also highlight the importance of CAR design; while CD28-co-stimulated CAR T cells had reduced alloreactivity, 4-1BB-co-stimulated and first-generation CAR T cells retained alloreactivity and increased risk of GVHD [83].

In a recent clinical trial, 8 r/r B-ALL patients received either HLA-matched (*n* = 4) or HLA-haploidentical (*n* = 4) CAR19T cells immediately preceding an intended HSCT as a leukemia debulking strategy [84]. The haploidentical CAR T cells induced transient or no reduction in peripheral blood leukemia with no significant CAR T cell expansion which suggests rejection [84]. Patients treated with HLA-matched CAR19T cells exhibited higher complete response rates although more severe toxic side effects compared with those treated with haploidentical CAR19T cells, with no GVHD observed in either group [84]. Only 3 out of 8 patients reached complete response and only 2 of the 8 patients proceeded to transplant, with all 4 haploidentical CAR19T-treated patients dying of disease progression and 1 HLA-matched CAR19T-treated patient dying of lung infection [84]. Thus HLA-matched and HLA-haploidentical allogeneic CD19-directed CAR T cell infusions are feasible in r/r B-ALL before HSCT, but other factors besides GVHD need to be considered in clinical applications of allogeneic CAR T cell infusions.

Interestingly, the generation of CAR T cells from hematopoietic stem cells (HSCs) may provide an avenue to overcome alloreactivity. Transgenic expression of CAR was shown to inhibit rearrangements of endogenous TCR during T cell differentiation from primitive HSCs [89]. Introducing CARs to HSCs or early T cell precursors should therefore provide only antigen-specific targeting while preventing non-specific allogeneic T cell activation [89–92]. We and others have shown that CD34^–^ CD7^+^ early T cell precursors with pro-T1 phenotype can be generated ex vivo from UCB-derived CD34^+^ stem cells using conditions that mimic the thymic microenvironment (OP9-DL1 cells) or feeder-free conditions and immobilized delta-like 4 (DL4) ligands [89–92]. We have shown that CAR19-transduced ex vivo generated CD34^–^ CD7^+^ T cell precursors can efficiently engraft in immunodeficient mice and generate mature T cells that express CAR19 [92, 93]. Delayed leukemia progression was seen in immunodeficient mice reconstituted with UCB-derived CAR19-modified T cell precursors and challenged with CD19^+^ leukemia cells in an allogeneic xenograft mouse model [92, 93]. Notably, CAR T cells generated from UCB-derived T cell precursors did not exhibit xenogeneic reactivity against the host in this model, suggesting that UCB-derived CAR T cell precursors can potentially be used in conjunction with HSCT [92].

Compared to PB-derived T cells, cord blood-derived T cells exhibit lower risk of GVHD but higher GVL activity [72, 83, 94]. However, the alloreactivity of UCB-derived CAR T cells must be ablated before they can be safely used as universal off-the-shelf CAR T cells, ready for administration into patients as needed. Since TCRs mediate alloreactivity, approaches to down-regulate TCR chain expression using RNA interference or removing endogenous TCRs from donor T cells using genome editing can be used [95].

Current clinical trials investigating the safety and efficacy of donor-derived CAR T cells against r/r MM (NCT04093596), r/r B cell leukemia or lymphoma (NCT03939026, NCT04416984, NCT03166878), or r/r T cell malignancies (NCT04264078) employ various strategies to minimize GVHD, and most, if not all, involve knocking out the *TRAC* and/or *B2M* genes [96–100]. Most CAR T cells are genetically engineered from αβ T cells, named for the disulfide-linked TCRα and TCRβ that form the TCR whose hypervariable or complementarity-determining regions recognize foreign antigens [101–105]. TCRα is formed via VJ recombination and TCRβ via VDJ recombination in early stages of T cell maturation to create the highly diverse repertoire of TCRs that recognize pathogenic and tumor antigens [106]. TCRαβ non-covalently associates with transmembrane protein heterodimers CD3δε and CD3γε to form a hexamer, which then associates with CD3ζζ to form the TCR-CD3 assembly or TCR complex (Fig. 1A) that enables intracellular signal transduction via phosphorylation of tyrosine residues in the immunoreceptor tyrosine-based activation motifs (ITAM) of CD3 chains [101–105]. The CD3ζζ homodimer is linked by a disulfide bond and CD3 proteins are essential for TCR surface expression [101–105]. Without CD3γ, CD3δ, or CD3ε, TCRαβ cannot leave the endoplasmic reticulum and is degraded [101–105]. Without CD3ζ, the TCRαβ-CD3δε-CD3γε hexamer is transported to lysosomal degradation rather than the cell surface [101–105]. Therefore, knocking down the expression of a single TCR chain using genome editing or non-genome editing methods can result in the loss of the whole TCR complex from the cell surface, creating TCR-knockout (TCR-KO) CAR T cells which can be isolated by screening for CD3^–^ CAR T cells. Recent proof-of-concept studies showed that the resulting TCR-KO CAR T cells do not respond to TCR stimulation but do respond to CAR stimulation [107]. Allogeneic anti-BCMA CAR T cells, generated using short hairpin RNA (shRNA) to target CD3ζ to knock out TCR, demonstrated systemic CAR T cell engraftment with no GVHD in a recent dose-escalation phase I clinical trial with 6 r/r MM patients [108, 109].

## Genome editing CAR T cells to enhance safety and efficacy

Genome editing can also be used to improve CAR T cell function and enhance antitumor responses to bypass tumor immune evasion strategies. Compared to other genome editing tools such as zinc finger nucleases (ZFNs) and transcription activator-like effector nucleases (TALENs), CRISPR-Cas shows much promise in generating universal CAR T cells due to its relative ease and reasonable costs [110]. Table 2 compares and summarizes the safety and efficacy of various gene-edited CAR T cells in recent clinical trials.

TALENs are restriction enzymes comprised of transcription activator-like effectors (TALEs), derived from proteins secreted by *Xanthomonas* spp. bacteria, fused to *Fok*I endonucleases via the C-terminal linker for targeted DNA cleavage [110–112]. *Fok*I endonucleases bind to the 5’-GGATG-3’ recognition site and cleave the 5’ strand 9 base pairs away and the 3’ strand 13 base pairs away [113]. TALEs consist of central DNA-binding domains flanked by N-terminal translocation domains for binding to the target DNA preferentially at 5’ thymine [114], and C-terminal activation domains with nuclear localization signals for translocation into cell nuclei [110–112]. Within the DNA-binding domains are repeated, highly conserved 33–34 amino acid sequences with divergent amino acids at positions 12 and 13, known as the repeat variable di-residue (RVD) [110–112]. RVDs are highly variable but generally recognize specific nucleotide bases: NI (asparagine, isoleucine) for adenosine, NG (asparagine, glycine) for thymine, HD (histidine, aspartic acid) for cytosine, and NN (asparagine, asparagine) preferentially for guanine [110–112]. Thus TALENs can be designed to recognize and cut any DNA sequence by combining segments that have the suitable RVDs (Fig. 1B). Double-strand breaks (DSB) induced by TALENs are repaired by either non-homologous end joining (NHEJ) or homology directed repair (HDR) mechanisms [110–112].

In a recently completed phase I clinical trial (NCT02808442) conducted by Qasim et al., human infants were treated with allogeneic CD19-specific CAR T cells generated via lentiviral transduction followed by TALEN-mediated disruption of TRAC and CD52 [57, 115, 116]. *TRAC* disruption prevented TCRαβ cell surface expression, and residual TCRαβ^+^ cells were removed using magnetic beads. CD19^+^ r/r B-ALL patients received lymphodepleting chemotherapy, anti-CD52 serotherapy (alemtuzumab), and 1 dose of TCR^–^ CD52^–^ CAR19 T cells that established molecular remission within 28 days without GVHD which persisted until conditioning before allo-SCT [57, 115, 116]. Antitumor activity and CAR T cell persistence was dependent on receipt of alemtuzumab conditioning [117]. Persistence of CAR T cells in the peripheral blood was seen up to 80 days post-infusion, but assessment of long-term persistence was limited due to the majority of patients proceeding to allogeneic stem cell transplant. Complete response or complete response with incomplete hematological recovery was seen in 67% patients and the 6-month progression-free survival was 27% [57, 115, 116]. Despite depleting TCR^+^ cells from the product, GVHD was seen in conjunction with expansion of CAR^+^ TCR^+^ T cells in 10% patients. While the results in B-ALL are inferior to those seen with autologous products, they provide a reference point for future products to improve upon [22, 53].

Clustered regularly interspaced short palindromic repeats (CRISPR) and CRISPR-associated (Cas) proteins are part of bacterial adaptive immunity against bacteriophage infections and work by digesting invading DNA [118–120]. Although many variants of CRISPR-Cas exist, the type II CRISPR-Cas9 system found in *Streptococcus pyogenes* is the simplest and most often used in genome editing (Fig. 1C). To cut DNA, the Cas9 endonuclease forms a ribonucleoprotein (RNP) complex with a synthetic single guide RNA (sgRNA) consisting of a CRISPR RNA (crRNA) joined to a trans-activating CRISPR RNA (tracrRNA) by a linker loop [118–120]. The crRNA provides the specificity for Cas9 and has a 20-nucleotide sequence complementary to the target sequence [118–120]. At the HNH and RuvC nuclease active sites in Cas9, blunt-ended double-strand breaks (DSB) are created in the target DNA approximately 3 nucleotides upstream of the protospacer adjacent motif (PAM, 5’-NGG-3’ for SpCas9 where N is any nucleotide) [118–120]. DSB are repaired by either NHEJ or HDR mechanisms (Fig. 1D). NHEJ is favored but error-prone, and creates insertions or deletions (indels) in target DNA. This results in frameshift mutations that disrupt gene function and can be used to knock out genes. HDR is more precise but requires single-stranded or double-stranded DNA donor templates containing genes of interest (GOI) flanked on both sides by homology arms (HA) that match the sequences next to the genomic target [118–120]. HDR is used to knock in genes since it replaces the target sequence with the sequence in the donor template. Genome editing of mammalian cells by CRISPR-Cas9 requires longer tracrRNA sequences and additional nuclear localization signals to enable Cas9 to access cell nuclei [118–120].

Pitfalls of genome editing include off-target mutations that can potentially be oncogenic depending on the sequences involved [121]. CRISPR-Cas9 induces DSB repair that can result in large deletions and complex rearrangements [121]. Partial mismatching is tolerated by Cas9 which can inadvertently allow cutting of DNA despite the match being several nucleotides different to the exact target sequence [121]. With mammalian DNA being much longer than the prokaryotic DNA that Cas originated in, chances of off-target effects are increased when editing mammalian cells [121]. CRISPR-Cas9 genome editing also induces p53-mediated DNA damage responses [122]. In human pluripotent stem cells (PSCs), p53 inhibits CRISPR-Cas9 engineering, leading to selection against cells with functional p53 pathways [122]. p53 inhibition prevents the damage response and increases the rate of HDR from donor templates. Taken together, the results suggest that CRISPR-Cas9 engineering in human cells may lead to accumulation of genome edited cells with dysfunctional p53 and increased risk of neoplastic transformation [122]. This, along with other clinical toxicities like CRS, prompts the need to carefully modulate CAR T cell levels for patient safety without reducing antitumor efficacy. One effective strategy is to incorporate pharmacologically inducible suicide genes into CAR constructs as safety switches; for example, inducible caspase-9 (*iC9* or *iCasp9*) which consists of fused domains modified from human caspase-9 and FK506-binding protein-12 (FKBP12) [123–126]. Small-molecule chemical inducers of dimerization (CID), such as rimiducid (AP1903) or the B/B homodimerizer (AP20187), can then be used to eliminate excess iC9-transduced CAR T cells by cross-linking FKBP domains to synthetically activate caspase-9 and initiating intrinsic apoptotic pathways for rapid cell death [123–126].

Although the issue of off-target mutagenesis has been largely improved due to recent advances in CRISPR-Cas base editing and prime editing, potential effects of off-target gene editing are still largely unknown and unpredictable, especially in approaches that target multiple genes at once [127–129]. Whole-genome sequencing is important to accurately assess off-target effects and develop algorithms for predicting single and multiplex off-target cleavage sites. One possible solution is to generate CAR T cells from PB-derived induced pluripotent stem cell (iPSC) lines that have been genome edited to enhance antitumor properties or modify TCR and HLA genes to reduce alloreactivity and graft rejection [130]. While iPSC lines can be screened and validated under GMP conditions before use, the lack of clinically compatible feeder-free or serum-free differentiation methods to generate enough mature T cells for subsequent CAR modification poses a significant challenge [130]. However, with extensive preclinical analyses to assess the safety and efficacy of genome edited anti-cancer immunotherapy products, banks of TCR-KO CAR T cells can be made in advance from common HLA-expressing blood donors for a broad cohort of patients and be readily used to treat hematologic cancers.

T-ALL comprises 20–25% of cases of all adult ALL and is notoriously difficult to treat due to its complexity and quick progression [131, 132]. CD7 is expressed on more than 95% of T-ALL but also expressed on normal T cells, which complicates the development of CD7-targeting CAR T cells due to potential contamination by T-ALL cells, and target-driven T cell fratricide which limits adequate CAR T cell production [131, 132]. It is encouraging that donor-derived anti-CD7 CAR T cells achieved efficient expansion with high complete remission rates and manageable safety profile in a recent phase I trial of 20 r/r T-ALL patients [131]. However, the value of genome-edited CAR T cells is demonstrated in another recent early-phase clinical trial of 5 adult r/r T-ALL patients where Universal CAR T therapy (TruUCART™ GC027) was shown to be safe and effective [132]. TruUCART™ GC027 was generated using lentiviral vectors to deliver second-generation CAR onto T cells sourced from HLA-mismatched healthy donors, with CRISPR-Cas9-mediated knockout of TCRα and CD7 to minimize GVHD and T cell fratricide [132]. Preclinical testing in CCRF-CEM xenograft mouse models showed strong antitumor activity and prolonged survival in all treated groups, and 80% of human patients treated with a single infusion of TruUCART™ GC027 without preconditioning showed robust CAR T cell expansion and persisting MRD^–^ complete responses [132]. Updates to this trial showed 5 out of 6 patients achieving MRD^–^ remission at 1 month which was maintained in 3 patients at 6 months [133]. The authors reported robust early expansion of the CAR T cells, but no information was provided regarding long-term persistence. Further promising results from Georgiadis et al. showed that base-edited CAR T cells exhibit no chromosomal translocations or off-target mutations that may affect CAR T cell specificity in preclinical studies using Jurkat and patient T-ALL cells and NSG mice [134]. Activated donor T cells were electroporated with sgRNA targeting TRBC and CD7 and codon optimized BE3 mRNA, followed by lentiviral transduction with 3CAR and 7CAR [134]. Precise multiplexed CRISPR base-editing was used to disrupt *TRBC1/2* and *CD7* to create TCRαβ/CD3^–^ and CD7^–^ CAR T cells, with shared antigens CD3 and CD7 removed to prevent T cell fratricide [134]. Fratricide-resistant TCR^–^ CD3^–^ CD7^–^ CAR T cells showed high antitumor activity against T-ALL targets in vitro and in vivo [134].

Other phase I clinical trials (NCT03399448) demonstrate the safety and feasibility of CRISPR-Cas9 multiplex editing [135, 136]. Adult patients (age 62–66 yrs) were treated with autologous T cells generated by electroporation of CRISPR-Cas9 RNP complexes (containing 3 sgRNAs targeting *TRAC, TRBC1, TRBC2, PDCD1*) and lentiviral transduction of NY-ESO-1 and LAGE-1 cancer-specific TCR into patient T cells [135, 136]. Multiplex CRISPR-Cas9 editing was used to delete TRAC, TRBC, and PD-1 encoding genes. Endogenous TCR were deleted before replacement with transgenic TCR (specific for NY-ESO-1 and LAGE-1) to minimize transgenic and endogenous mixed-dimer formation. Endogenous PD-1 knockout enhances T cell persistence and antitumor immunity [135, 136]. Durable engraftment of transgenic T cells was achieved with genomic edits at all 3 loci and minimal chromosomal translocations that decreased after infusion into patients. T cells trafficked to tumor sites and persisted for 9 months with minimal immunogenicity, and biopsies showed residual tumors but reduced NY-ESO-1 and/or LAGE-1 in refractory myeloma patients [135, 136].

Another important application of multi-targeted gene editing is in creating CAR T cells that mitigate the antigen escape responsible for CD19^–^ relapses in B cell malignancies. A potential strategy is to also target CD22, which is highly expressed by lymphoid blasts in 60–90% of B-ALL [137]. Hu et al. showed this in a dose-escalation phase I clinical trial on adult r/r ALL patients treated with universal CD19/CD22 dual-targeting CAR T cells (CTA101) [138]. Activated CD3^+^ T cells were transduced with lentiviral constructs consisting of CD19 and CD22 scFv with 4-1BB co-stimulatory and CD3ζ signaling domains, followed by electroporation for CRISPR-Cas9 mediated knockout of *TRAC* and *CD52* genes and depletion of TCR/CD3^+^ cells [138]. Patients treated with CTA101 did not exhibit GVHD, neurotoxicity, or gene editing associated toxicities, but suffered from manageable levels of CRS [138]. CTA101 demonstrated robust anti-leukemic activity with 83.3% of patients achieving complete remission 28 days post-treatment, and 60% of these patients remained MRD^–^ 4.3 months post-treatment [138].

As more genomic tools become available, CAR constructs can be further refined by testing combinations of features in preclinical studies. Experiments by Eyquem et al. on CD19^+^ cell lines and NSG mice demonstrated that, compared to conventional CAR T cells, *TRAC*-CAR T cells had uniform CAR expression and higher anti-leukemic potency in vivo [139]. Activated T cells were electroporated with Cas9 mRNA and guide RNA (gRNA) and then transduced with rAAV6 (containing the CAR cassette, *TRAC*-1928z, flanked by HA) for CRISPR/Cas9 mediated CAR19 gene knock-in to the *TRAC* locus. Integration of CAR into the *TRAC* locus mitigates tonic signaling, promotes CAR expression on antigen exposure, and delays effector T cell differentiation and exhaustion [139].

Kagoya et al. tested triple knockout (tKO) CAR T cells in leukemia and melanoma cell lines and in NSG mice [140]. T cells were electroporated with Cas9/sgRNA RNPs and retrovirally transduced with CAR19 to generate CRISPR-Cas9 mediated HLA-I, HLA-II, and TCR tKO CAR T cells (using targeted sgRNA to simultaneously knock out *B2M, CIITA,* and *TRAC* genes) [140]. After expansion, HLA and TCR KO cells were isolated using FACS or magnetic beads. Multiplexed gene KO did not affect CAR T cell function and tKO CAR19 T cells had high anti-leukemic activity but did not induce GVHD [140]. Deletion of HLA-II, TCR, and B2M abrogated alloreactivity in tKO CAR T cells [140]. It is relevant that both HLA-I and HLA-II needed to be ablated for efficient donor T cell persistence. Compared with TRAC and B2M double-KO T cells, tKO CAR T cells retained antitumor responses, showed better persistence, and did not exhibit alloreactivity when cultured with allogeneic PBMCs [140]. These results demonstrate the benefits of HLA-I, HLA-II, and TCR deletion for enabling donor T cells to be used as off-the-shelf adoptive immunotherapy [140].

Unlike the previous studies, Kamiya et al. did not use genome editing but instead used anti-CD3ε protein expression blockers (PEBLs) to block surface expression of CD3/TCRαβ [141]. MSCV retroviral vectors containing anti-CD19-41BB-CD3ζ and PEBL constructs and mRNA were electroporated into T cells [141]. Compared to CD3/TCRαβ^+^ CAR T cells, anti-CD3ε PEBL CAR T cells induced similar or higher cytokine secretion, proliferation, and anti-leukemic activity but with greatly reduced xenogeneic GVHD potential in NSG mice [141].

The general approach of removing HLA or TRAC expression in CAR T cells described above demonstrates promising results in reducing GVHD while maintaining high antitumor efficacy [139–141]. However, this may not be the case depending on the method used to remove TCR expression, as shown by Stenger et al. who used CRISPR-Cas9 mediated KO of endogenous TCRβ to create TCR^–^ CAR T cells [142]. T cells were retrovirally transduced with second-generation CAR19 (containing CD8 transmembrane and 4-1BB co-stimulatory domains), then electroporated with Cas9/gRNA RNP [142]. These TCR^–^ CAR T cells showed strong activation and proliferation with significantly lower alloreactivity but shorter persistence and lower anti-leukemic activity than TCR^+^ CAR T cells [142]. Our recent experiments with transposon-based TCR^–^ CAR19 T cells, generated via CRISPR-Cas9-mediated CD3γ knockout, showed equally high antitumor activity but lesser persistence than TCR^+^ CAR19 T cells based on the same construct (unpublished data). This may lead to reduced duration of remission induced by TCR^–^ CAR T cells compared to TCR^+^ CAR T cells, and aligns with the reduced persistence reported by Stenger et al. [142].

Interestingly, Roth et al. showed that non-viral multiplexed genome editing can also provide a fast, simple, and cost-effective method of engineering T cells [143]. Human T cells were co-electroporated with CRISPR-Cas9 RNP and dsDNA HDR templates for non-viral CRISPR-Cas9 genome targeting of endogenous *TRAC* exon 1 on T cells, replacing it by integrating HDR templates with NY-ESO-1 antigen-specific TCR [143]. NY-ESO-1 TCR knock-in T cells trafficked to tumors, where they persisted and proliferated to produce effective antitumor responses comparable to lentiviral-transduced T cells in NSG mice and in melanoma cell lines [143].

Modifications by genome editing on the genomic level provide opportunities to modulate inhibitory signals to enhance antitumor effects. Initial results have shown that multiple genomic modifications of T cells are feasible, and it is expected that this will lead to more multiplexed genome-edited anti-cancer cellular products. However, there is still much to be done before allogeneic CAR T cells can fully replace autologous CAR T cells, since most of the current clinical trials to confirm high efficacy and long-term safety are done with autologous cellular therapies [1–19]. Despite encouraging results, most studies with sophisticated allogeneic products are still in the preclinical stage due to design and/or production challenges, particularly those manufactured using non-viral vectors or involving genome editing, since potential oncogenicity or off-target mutagenesis must first be eliminated before progression to clinical use. An example is the FDA’s recent temporary hold on several phase I/II AlloCAR T clinical trials: ALPHA [98], ALPHA-2 [100], IGNITE [144], TRAVERSE [145], and UNIVERSAL [97]. An abnormality on chromosome 14 (location of the *TRAC* locus) was detected in the bone marrow biopsy of a patient treated with ALLO-501A CAR T cells during the ALPHA-2 trial [146, 147]. Testing is needed to determine if the abnormality arose from the gene edits, if this presents a risk for other CAR T cells that use similar gene editing techniques, and how effects may change as CAR T cell levels rise then drop during the course of therapy [146, 147]. The FDA clinical hold has since been lifted from all AlloCAR T clinical trials, since the chromosomal abnormality was clinically insignificant and only occurred in this particular patient due to rearrangement of TCR and immunoglobulin gene regions during T or B cell maturation, and not due to the AlloCAR T manufacture process or TALEN gene editing [148].

## Conclusion

T cell immunotherapy using donor T cells appears promising in aiming for better disease control in patients with malignant indications. However, improvements are needed before it can be developed into standard therapeutics. Applications of genome editing techniques to donor T cells should be explored for safety, feasibility, and whether it can lead to better next-generation treatments for hematologic malignancies and other cancers.

## Data Availability

Data sharing not applicable to this article as no datasets were generated or analyzed during the current study.

## Abbreviations

AAV6: adeno-associated virus serotype 6
AdV: adenovirus
AR: antigen receptor
AUC: area under the curve
B2M: beta-2 microglobulin
BCMA: B cell maturation antigen
B-ALL: B cell acute lymphoblastic leukemia
CAR: chimeric antigen receptor
CAR19: anti-CD19 chimeric antigen receptor
Cas: CRISPR-associated
CCR7: C–C chemokine receptor type 7
CD: cluster of differentiation
CID: chemically induced dimerization
CIITA: class II major histocompatibility complex transactivator
CLL: chronic lymphocytic leukemia
CMV: cytomegalovirus
CR: complete response
CRES: CAR T cell associated encephalopathy syndrome
CRISPR: clustered regularly interspaced short palindromic repeats
crRNA: CRISPR RNA
CRS: cytokine release syndrome
CTAG1B: cancer-testis antigen 1B
dKO: double knockout
DLBCL: diffuse large B cell lymphoma
DSB: double-strand break
dsDNA: double-stranded DNA
EBV: Epstein-Barr virus
FACS: fluorescence-activated cell sorting
FKBP12: FK506-binding protein-12
FL: follicular lymphoma
GMP: good manufacturing practice
GOI: gene of interest
gRNA: guide RNA
GVHD: graft versus host disease
GVL: graft versus lymphoma
HA: homology arm
haplo-HSCT: haploidentical hematopoietic stem cell transplantation
HDR: homology directed repair
HLA: human leukocyte antigen
HPC: hematopoietic progenitor cell
HSC: hematopoietic stem cell
HSCT: hematopoietic stem cell transplantation
iC9: inducible caspase-9 (aka iCasp9)
ICANS: immune effector cell-associated neurotoxicity syndrome
IFN: interferon
IL: interleukin
IL2RA: interleukin-2 receptor alpha chain
indel: insertion/deletion polymorphism
iPSC: induced pluripotent stem cell
ITAM: immunoreceptor tyrosine-based activation motif
KO: knockout
LAGE-1: L antigen family member 1 (aka NY-ESO-2)
LBCL: large B cell lymphoma
LCL: lymphoblastoid cell line
MCL: mantle cell lymphoma
MM: multiple myeloma
MRD: minimal residual disease
MSCV: murine stem cell virus
MUD: matched unrelated donor
NHEJ: non-homologous end joining
NHL: non-Hodgkin lymphoma
NSG: NOD *scid** gamma* (NOD.*Cg-Prkdc*^*scid*^*Il2rg*^*tm1Wjl*^*/*SzJ)
nt: nucleotide
NY-ESO-1: New York esophageal squamous cell carcinoma-1 (aka CTAG1B)
ORR: overall response rate
PAM: protospacer adjacent motif
PB: peripheral blood
PBMC: peripheral blood mononuclear cell
PD-1: programmed cell death protein 1
PD-L1: programmed death-ligand 1
PDX: patient-derived xenograft
PEBLs: protein expression blockers
PR: partial response
PTCy: post-transplant cyclophosphamide
PSC: pluripotent stem cells
r/r: relapsed or refractory
rAAV6: recombinant adeno-associated virus serotype 6
RNP: ribonucleoprotein
RVD: repeat variable di-residue
scFv: single chain variable fragment
SCT: stem cell transplantation
sgRNA: single guide RNA
shRNA: short‑hairpin RNA
SpCas9: *Streptococcus pyogenes* Cas9
ssDNA: single-stranded DNA
TALENs: transcription activator-like effector nucleases
TALEs: transcription activator-like effectors
T-ALL: T cell acute lymphoblastic leukemia
TCL: T cell lymphoma
TCR: T cell receptor
tgTCR: transgenic T cell receptor
tKO: triple knockout
TM: transmembrane domain
TNF: tumor necrosis factor
TRAC: T cell receptor alpha constant chain
tracrRNA: trans-activating CRISPR RNA
TRBC: T cell receptor beta constant chain
T_SCM_: T stem cell memory
UCB: umbilical cord blood
VH: variable heavy
VL: variable light
ZFNs: zinc finger nucleases

## References

[1] Berdeja JG, Madduri D, Usmani SZ, Jakubowiak A, Agha M, Cohen AD (2021). Ciltacabtagene autoleucel, a B-cell maturation antigen-directed chimeric antigen receptor T-cell therapy in patients with relapsed or refractory multiple myeloma (CARTITUDE-1): a phase 1b/2 open-label study. Lancet.

[2] Hirayama AV, Gauthier J, Hay KA, Voutsinas JM, Wu Q, Pender BS (2019). High rate of durable complete remission in follicular lymphoma after CD19 CAR-T cell immunotherapy. Blood.

[3] Jacobson CA, Chavez JC, Sehgal AR, William BM, Munoz J, Salles GA (2020). Interim analysis of ZUMA-5: A phase II study of axicabtagene ciloleucel (axi-cel) in patients (pts) with relapsed/refractory indolent non-Hodgkin lymphoma (R/R iNHL). J Clin Oncol.

[4] Lemal R, Tournilhac O (2019). State-of-the-art for CAR T-cell therapy for chronic lymphocytic leukemia in 2019. J Immunother Cancer.

[5] Locke FL, Ghobadi A, Jacobson CA, Miklos DB, Lekakis LJ, Oluwole OO (2019). Long-term safety and activity of axicabtagene ciloleucel in refractory large B-cell lymphoma (ZUMA-1): a single-arm, multicentre, phase 1–2 trial. Lancet Oncol.

[6] Locke FL, Neelapu SS, Bartlett NL, Siddiqi T, Chavez JC, Hosing CM (2017). Phase 1 Results of ZUMA-1: A Multicenter Study of KTE-C19 Anti-CD19 CAR T Cell Therapy in Refractory Aggressive Lymphoma. Mol Ther.

[7] Munshi NC, Anderson LD, Shah N, Madduri D, Berdeja Js, Lonial S (2021). Idecabtagene vicleucel in relapsed and refractory multiple myeloma. N Eng J Med.

[8] Nastoupil LJ, Jain MD, Feng L, Spiegel JY, Ghobadi A, Lin Y (2020). Standard-of-Care Axicabtagene Ciloleucel for Relapsed or Refractory Large B-Cell Lymphoma: Results From the US Lymphoma CAR T Consortium. J Clin Oncol.

[9] Pehlivan KC, Duncan BB, Lee DW (2018). CAR-T Cell Therapy for Acute Lymphoblastic Leukemia: Transforming the Treatment of Relapsed and Refractory Disease. Curr Hematol Malig Rep.

[10] Schuster SJ, Bishop MR, Tam CS, Waller EK, Borchmann P, McGuirk JP (2019). Tisagenlecleucel in Adult Relapsed or Refractory Diffuse Large B-Cell Lymphoma. N Engl J Med.

[11] Shah BD, Bishop MR, Oluwole OO, Logan AC, Baer MR, Donnellan WB (2021). KTE-X19 anti-CD19 CAR T-cell therapy in adult relapsed/refractory acute lymphoblastic leukemia: ZUMA-3 phase 1 results. Blood.

[12] Shah BD, Ghobadi A, Oluwole OO, Logan A, Boissel N, Cassaday RD (2021). Phase 2 results of the ZUMA-3 study evaluating KTE-X19, an anti-CD19 chimeric antigen receptor (CAR) T-cell therapy, in adult patients (pts) with relapsed/refractory B-cell acute lymphoblastic leukemia (R/R B-ALL). J Clin Oncol.

[13] Shah BD, Ghobadi A, Oluwole OO, Logan AC, Boissel N, Cassaday RD (2021). KTE-X19 for relapsed or refractory adult B-cell acute lymphoblastic leukaemia: phase 2 results of the single-arm, open-label, multicentre ZUMA-3 study. The Lancet.

[14] Teoh PJ, Chng WJ (2021). CAR T-cell therapy in multiple myeloma: more room for improvement. Blood Cancer J.

[15] Vitale C, Strati P (2020). CAR T-Cell therapy for b-cell non-hodgkin lymphoma and chronic lymphocytic leukemia: clinical trials and real-world experiences. Front oncol.

[16] Wang M, Locke FL, Munoz J, Goy A, Holmes HE, Siddiqi T (2018). ZUMA-2: Phase 2 multicenter study evaluating efficacy of kte-C19 in patients with relapsed/refractory mantle cell lymphoma. J Clin Oncol.

[17] Wang M, Munoz J, Goy A, Locke FL, CAJacobson, Hill BT (2021). Outcomes with KTE-X19 in patients (pts) with relapsed/refractory (R/R) mantle cell lymphoma (MCL) in ZUMA-2 who had progression of disease within 24 months of diagnosis (POD24). J Clin Oncol.

[18] Wang M, Munoz J, Goy A, Locke FL, Jacobson CA, Hill BT (2020). KTE-X19 CAR T-Cell Therapy in Relapsed or Refractory Mantle-Cell Lymphoma. N Engl J Med.

[19] Yang C, Lei W, Xie H, Wu G, Wei J, Liang A (2020). Sustained Remission of Relapsed or Refractory Mantle Cell Lymphoma After 4–1BB-Based CD19-Directed CAR-T Therapy. Onco Targets Ther.

[20] Brentjens RJ, Davila ML, Riviere I, Park J, Wang X, Cowell LG (2013). CD19-targeted T cells rapidly induce molecular remissions in adults with chemotherapy-refractory acute lymphoblastic leukemia. Sci Transl Med.

[21] Brentjens RJ, Rivière I, Park JH, Davila ML, Wang X, Stefanski J (2011). Safety and persistence of adoptively transferred autologous CD19-targeted T cells in patients with relapsed or chemotherapy refractory B-cell leukemias. Blood.

[22] Gardner RA, Finney O, Annesley C, Brakke H, Summers C, Leger K (2017). Intent-to-treat leukemia remission by CD19 CAR T cells of defined formulation and dose in children and young adults. Blood.

[23] Lee DW, Kochenderfer JN, Stetler-Stevenson M, Cui YK, Delbrook C, Feldman SA (2015). T cells expressing CD19 chimeric antigen receptors for acute lymphoblastic leukaemia in children and young adults: a phase 1 dose-escalation trial. Lancet.

[24] Maude SL, Frey N, Shaw PA, Aplenc R, Barrett DM, Bunin NJ (2014). Chimeric antigen receptor T cells for sustained remissions in leukemia. N Engl J Med.

[25] Neelapu SS, Locke FL, Bartlett NL, Lekakis LJ, Miklos DB, Jacobson CA (2017). Axicabtagene Ciloleucel CAR T-Cell Therapy in Refractory Large B-Cell Lymphoma. N Engl J Med.

[26] FDA. KYMRIAH (tisagenlecleucel): U.S. Food & Drug Administration; 2021 [updated Jun 14, 2021. Available from: https://www.fda.gov/vaccines-blood-biologics/cellular-gene-therapy-products/kymriah-tisagenlecleucel.

[27] FDA. YESCARTA (axicabtagene ciloleucel): U.S. Food & Drug Administration; 2021 [updated May 11, 2021. Available from: https://www.fda.gov/vaccines-blood-biologics/cellular-gene-therapy-products/yescarta-axicabtagene-ciloleucel.

[28] FDA. TECARTUS (brexucabtagene autoleucel): U.S. Food & Drug Administration; 2021 [updated Mar 18, 2021. Available from: https://www.fda.gov/vaccines-blood-biologics/cellular-gene-therapy-products/tecartus-brexucabtagene-autoleucel.

[29] FDA. ABECMA (idecabtagene vicleucel): U.S. Food & Drug Administration; 2021 [updated Apr 21, 2021. Available from: https://www.fda.gov/vaccines-blood-biologics/cellular-gene-therapy-products/kymriah-tisagenlecleucel.

[30] Guedan S, Calderon H, Posey AD, Maus MV (2018). Engineering and Design of Chimeric Antigen Receptors. Mol Ther Methods Clin Dev.

[31] Sadelain M, Brentjens R, Riviere I (2013). The basic principles of chimeric antigen receptor design. Cancer Discov.

[32] Tyagarajan S, Spencer T, Smith J (2020). Optimizing CAR-T Cell Manufacturing Processes during Pivotal Clinical Trials. Molecular Therapy: Methods & Clinical Development.

[33] Mock U, Nickolay L, Philip B, Cheung GW, Zhan H, Johnston ICD (2016). Automated manufacturing of chimeric antigen receptor T cells for adoptive immunotherapy using CliniMACS prodigy. Cytotherapy.

[34] Agarwal S, Hanauer JDS, Frank AM, Riechert V, Thalheimer FB, Buchholz CJ (2020). In Vivo Generation of CAR T Cells Selectively in Human CD4+ Lymphocytes. Mol Ther.

[35] Pfeiffer A, Thalheimer FB, Hartmann S, Frank AM, Bender RR, Danisch S (2018). In vivo generation of human CD19-CAR T cells results in B-cell depletion and signs of cytokine release syndrome. EMBO Mol Med..

[36] Nawaz W, Huang B, Xu S, Li Y, Zhu L, Yiqiao H (2021). AAV-mediated in vivo CAR gene therapy for targeting human T-cell leukemia. Blood Cancer J.

[37] Bishop DC, Xu N, Tse B, O'Brien TA, Gottlieb DJ, Dolnikov A (2018). PiggyBac-Engineered T Cells Expressing CD19-Specific CARs that Lack IgG1 Fc Spacers Have Potent Activity against B-ALL Xenografts. Mol Ther.

[38] ANZCTR. A Phase I Study of CD19 Specific Chimeric Antigen Receptor T-cells for Therapy of Persistent and Relapsed B-cell Leukaemia and Lymphoma Post Allogeneic Stem Cell Transplantation (CARTELL) (Registry ID: ACTRN12617001579381) Sydney, Australia: National Health and Medical Research Council Australia; 2018 [updated Nov 1, 2018. Available from: https://www.anzctr.org.au/Trial/Registration/TrialReview.aspx?id=373934.

[39] ClinicalTrials.gov. CD19-specific CAR-T Cells in CLL/SLL and DLBCL (Registry ID: NCT03960840): Novartis Pharmaceuticals; 2019 [updated May 14, 2021. Available from: https://ClinicalTrials.gov/show/NCT03960840.

[40] ClinicalTrials.gov. Anti-CD19 CAR in PiggyBac Transposon-Engineered T Cells for Relapsed/Refractory B-cell Lymphoma or B-cell Acute Lymphoblastic Leukemia (Registry ID: NCT04289220): Yan'an Affiliated Hospital of Kunming Medical University; 2020 [updated May 13, 2021. Available from: https://ClinicalTrials.gov/show/NCT04289220.

[41] Bishop DC, Clancy LE, Simms R, Burgess J, Mathew G, Moezzi L, Street JA, Sutrave G, Atkins E, McGuire HM, Gloss BS, Lee K, Jiang W, Maddock K, McCaughan G, Avdic S, Antonenas V, O'Brien TA, Shaw PJ, Irving DO, Gottlieb DJ, Blyth E, Micklethwaite KP. Development of CAR T-cell lymphoma in 2 of 10 patients effectively treated with piggyBac-modified CD19 CAR T cells. Blood. 2021;138(16):1504–9. 10.1182/blood.2021010813. 10.1182/blood.2021010813 34010392

[42] Micklethwaite KP, Gowrishankar K, Gloss BS, Li Z, Street JA, Moezzi L, Mach MA, Sutrave G, Clancy LE, Bishop DC, Louie RHY, Cai C, Foox J, MacKay M, Sedlazeck FJ, Blombery P, Mason CE, Luciani F, Gottlieb DJ, Blyth E. Investigation of product-derived lymphoma following infusion of piggyBac-modified CD19 chimeric antigen receptor T cells. Blood. 2021;138(16):1391–405. 10.1182/blood.2021010858. 10.1182/blood.2021010858 PMC8532197 33974080

[43] ClinicalTrials.gov. Determine Efficacy and Safety of CTL019 in Pediatric Patients With Relapsed and Refractory B-cell ALL and High Risk B-cell ALL at First Relapse. Determine Feasibility and Safety of CTL019 Therapy in Pediatric Patients With High Risk B-cell ALL That Relapsed < 6 Months Post All-HSCT. (ELIANA) (Registry ID: NCT02435849) 2015 [updated June 15, 2021. Available from: https://ClinicalTrials.gov/show/NCT02435849.

[44] Grupp SA, Maude SL, Rives S, Baruchel A, Boyer MW, Bittencourt H (2018). Updated Analysis of the Efficacy and Safety of Tisagenlecleucel in Pediatric and Young Adult Patients with Relapsed/Refractory (r/r) Acute Lymphoblastic Leukemia. Blood.

[45] Fagnoni FF, Lozza L, Zibera C, Zambelli A, Ponchio L, Gibelli N (2002). T-cell dynamics after high-dose chemotherapy in adults: elucidation of the elusive CD8+ subset reveals multiple homeostatic T-cell compartments with distinct implications for immune competence. Immunology.

[46] Hakim FT, Cepeda R, Kaimei S, Mackall CL, McAtee N, Zujewski J (1997). Constraints on CD4 Recovery Postchemotherapy in Adults: Thymic Insufficiency and Apoptotic Decline of Expanded Peripheral CD4 Cells. Blood.

[47] Mackall CL, Fleisher TA, Brown MR, Andrich MP, Chen CC, Feuerstein IM (1997). Distinctions between CD8+ and CD4+ T-cell regenerative pathways result in prolonged T-cell subset imbalance after intensive chemotherapy. Blood.

[48] Verma R, Foster RE, Horgan K, Mounsey K, Nixon H, Smalle N (2016). Lymphocyte depletion and repopulation after chemotherapy for primary breast cancer. Breast Cancer Res.

[49] Lin H, Cheng J, Mu W, Zhou J, Zhu L. Advances in Universal CAR-T Cell Therapy. Front Immunol. 2021;12:744823. 10.3389/fimmu.2021.744823. 10.3389/fimmu.2021.744823 PMC8526896 34691052

[50] Caldwell KJ, Gottschalk S, Talleur AC (2021). Allogeneic CAR Cell Therapy-More Than a Pipe Dream. Front Immunol..

[51] June CH, O'Connor RS, Kawalekar OU, Ghassemi S, Milone MC (2018). CAR T cell immunotherapy for human cancer. Science.

[52] Depil S, Duchateau P, Grupp SA, Mufti G, Poirot L (2020). 'Off-the-shelf' allogeneic CAR T cells: development and challenges. Nat Rev Drug Discov.

[53] Rivers J, Annesley C, Summers C, Finney O, Pulsipher MA, Wayne AS (2018). Early response data for pediatric patients with Non-Hodgkin Lymphoma treated with CD19 chimeric antigen receptor (CAR) T-Cells. Blood.

[54] Costello C, Derman BA, Kocoglu MH, Deol A, Ali AA, Gregory T, et al. Abstract 3858: Clinical Trials of BCMA-Targeted CAR-T Cells Utilizing a Novel Non-Viral Transposon System. American Society of Hematology: 63rd ASH Annual Meeting & Exposition; 2021. https://ash.confex.com/ash/2021/webprogram/Paper151672.html.

[55] Costello CL, Cohen AD, Patel KK, Ali SS, Berdeja JG, Shah N, et al. Abstract 134: Phase 1/2 Study of the Safety and Response of P-BCMA-101 CAR-T Cells in Patients with Relapsed/Refractory (r/r) Multiple Myeloma (MM) (PRIME) with Novel Therapeutic Strategies. American Society of Hematology: 62rd ASH Annual Meeting & Exposition; 2020. https://ash.confex.com/ash/2020/webprogram/Paper142695.html.

[56] Costello CL, Gregory TK, Ali SA, Berdeja JG, Patel KK, Shah ND (2019). Phase 2 Study of the Response and Safety of P-Bcma-101 CAR-T Cells in Patients with Relapsed/Refractory (r/r) Multiple Myeloma (MM) (PRIME). Blood.

[57] Benjamin R, Graham C, Yallop D, Jozwik A, Mirci-Danicar OC, Lucchini G (2020). Genome-edited, donor-derived allogeneic anti-CD19 chimeric antigen receptor T cells in paediatric and adult B-cell acute lymphoblastic leukaemia: results of two phase 1 studies. Lancet.

[58] McGuirk J, Bachier CR, Bishop MR, Ho PJ, Murthy HS, Dickinson MJ (2021). A phase 1 dose escalation and cohort expansion study of the safety and efficacy of allogeneic CRISPR-Cas9-engineered T cells (CTX110) in patients (Pts) with relapsed or refractory (R/R) B-cell malignancies (CARBON). J Clin Oncol.

[59] CRISPR Therapeutics. [Press Release] CRISPR Therapeutics Reports Positive Results from its Phase 1 CARBON Trial of CTX110™ in Relapsed or Refractory CD19+ B-cell malignancies: Allogene Therapeutics; 2021 [updated Oct 12, 2021. Available from: http://www.crisprtx.com/about-us/press-releases-and-presentations/crispr-therapeutics-reports-positive-results-from-its-phase-1-carbon-trial-of-ctx110-in-relapsed-or-refractory-cd19-b-cell-malignancies.

[60] Alcantara M, Tesio M, June CH, Houot R (2018). CAR T-cells for T-cell malignancies: challenges in distinguishing between therapeutic, normal, and neoplastic T-cells. Leukemia.

[61] Fleischer LC, Spencer HT, Raikar SS (2019). Targeting T cell malignancies using CAR-based immunotherapy: challenges and potential solutions. J Hematol Oncol.

[62] Ruella M, Xu J, Barrett DM, Fraietta JA, Reich TJ, Ambrose DE (2019). Induction of resistance to chimeric antigen receptor T cell therapy by transduction of a single leukemic B cell. Nat Med.

[63] Hunger SP, Mullighan CG (2015). Acute Lymphoblastic Leukemia in Children. N Engl J Med.

[64] Marks DI, Rowntree C (2016). Management of adults with T-cell lymphoblastic leukemia. Blood.

[65] Dogan A, Morice WG (2004). Bone marrow histopathology in peripheral T-cell lymphomas. Br J Haematol.

[66] Marks DI, Paietta EM, Moorman AV, Richards SM, Buck G, DeWald G (2009). T-cell acute lymphoblastic leukemia in adults: clinical features, immunophenotype, cytogenetics, and outcome from the large randomized prospective trial (UKALL XII/ECOG 2993). Blood.

[67] Asnafi V, Beldjord K, Boulanger E, Comba B, Le Tutour P, Estienne MH (2003). Analysis of TCR, pT alpha, and RAG-1 in T-acute lymphoblastic leukemias improves understanding of early human T-lymphoid lineage commitment. Blood.

[68] Kochenderfer JN, Dudley ME, Carpenter RO, Kassim SH, Rose JJ, Telford WG (2013). Donor-derived CD19-targeted T cells cause regression of malignancy persisting after allogeneic hematopoietic stem cell transplantation. Blood.

[69] Kochenderfer JN, Somerville RPT, Lu T, Yang JC, Sherry RM, Feldman SA (2017). Long-Duration Complete Remissions of Diffuse Large B Cell Lymphoma after Anti-CD19 Chimeric Antigen Receptor T Cell Therapy. Mol Ther.

[70] Zhang C, Gao L, Liu Y, Gao L, Kong P-Y, Liu J (2019). Role of donor-derived CD19.CAR-T cells in treating patients that relapsed after allogeneic hematopoietic stem cell transplantation. Blood.

[71] Frumento G, Zheng Y, Aubert G, Raeiszadeh M, Lansdorp PM, Moss P (2013). Cord blood T cells retain early differentiation phenotype suitable for immunotherapy after TCR gene transfer to confer EBV specificity. Am J Transplant.

[72] Hiwarkar P, Qasim W, Ricciardelli I, Gilmour K, Quezada S, Saudemont A (2015). Cord blood T cells mediate enhanced antitumor effects compared with adult peripheral blood T cells. Blood.

[73] Yun HD, Varma A, Hussain MJ, Nathan S, Brunstein C. Clinical Relevance of Immunobiology in Umbilical Cord Blood Transplantation. J Clin Med. 2019;8(11):1968. 10.3390/jcm8111968. 10.3390/jcm8111968 PMC6912281 31739455

[74] Xu N, Tse B, Yang L, Tang TCY, Haber M, Micklethwaite K (2021). Priming Leukemia with 5-Azacytidine Enhances CAR T Cell Therapy. Immunotargets Ther.

[75] Cruz CRY, Micklethwaite KP, Savoldo B, Ramos CA, Lam S, Ku S (2013). Infusion of donor-derived CD19-redirected-virus-specific T cells for B-cell malignancies relapsed after allogeneic stem cell transplant: a phase i study. Blood.

[76] ClinicalTrials.gov. Infusion of Allogeneic Umbilical Cord Blood-Derived Cluster of Differentiation Antigen 19 (CD19)-Specific T Cells (Registry ID: NCT01362452): M.D. Anderson Cancer Center; 2017 [updated Aug 3, 2017. Available from: https://ClinicalTrials.gov/show/NCT01362452.

[77] Bleakley M, Turtle CJ, Riddell SR (2012). Augmentation of anti-tumor immunity by adoptive T-cell transfer after allogeneic hematopoietic stem cell transplantation. Expert Rev Hematol.

[78] Klamer G, Shen S, Song E, Rice AM, Knight R, Lindeman R (2013). GSK3 inhibition prevents lethal GVHD in mice. Exp Hematol.

[79] Wagner JE, Ballen KK, Zhang M-J, Allbee-Johnson M, Karanes C, Milano F (2021). Comparison of haploidentical and umbilical cord blood transplantation after myeloablative conditioning.

[80] Gooptu M, Romee R, St Martin A, Arora M, Al Malki M, Antin JH (2021). HLA-haploidentical vs matched unrelated donor transplants with posttransplant cyclophosphamide-based prophylaxis. Blood.

[81] Anwer F, Shaukat A-A, Zahid U, Husnain M, McBride A, Persky D (2017). Donor origin CAR T cells: graft versus malignancy effect without GVHD, a systematic review. Immunotherapy.

[82] Brudno JN, Somerville RP, Shi V, Rose JJ, Halverson DC, Fowler DH (2016). Allogeneic T Cells That Express an Anti-CD19 Chimeric Antigen Receptor Induce Remissions of B-Cell Malignancies That Progress After Allogeneic Hematopoietic Stem-Cell Transplantation Without Causing Graft-Versus-Host Disease. J Clin Oncol.

[83] Ghosh A, Smith M, James SE, Davila ML, Velardi E, Argyropoulos KV (2017). Donor CD19 CAR T cells exert potent graft-versus-lymphoma activity with diminished graft-versus-host activity. Nat Med.

[84] Jin X, Cao Y, Wang L, Sun R, Cheng L, He X (2020). HLA-matched and HLA-haploidentical allogeneic CD19-directed chimeric antigen receptor T-cell infusions are feasible in relapsed or refractory B-cell acute lymphoblastic leukemia before hematopoietic stem cell transplantation. Leukemia.

[85] Liu J, Zhong JF, Zhang X, Zhang C (2017). Allogeneic CD19-CAR-T cell infusion after allogeneic hematopoietic stem cell transplantation in B cell malignancies. J Hematol Oncol.

[86] Cruz CRY, Micklethwaite KP, Savoldo B, Ramos CA, Lam S, Ku S (2013). Infusion of donor-derived CD19-redirected virus-specific T cells for B-cell malignancies relapsed after allogeneic stem cell transplant: a phase 1 study. Blood.

[87] Zhang C, Wang X-Q, Zhang R-L, Liu F, Wang Y, Yan Z-L (2021). Donor-derived CD19 CAR-T cell therapy of relapse of CD19-positive B-ALL post allotransplant. Leukemia.

[88] Pan J, Tan Y, Wang G, Deng B, Ling Z, Song W (2021). Donor-Derived CD7 Chimeric Antigen Receptor T Cells for T-Cell Acute Lymphoblastic Leukemia: First-in-Human, Phase I Trial. J Clin Oncol.

[89] Van Caeneghem Y, De Munter S, Tieppo P, Goetgeluk G, Weening K, Verstichel G (2017). Antigen receptor-redirected T cells derived from hematopoietic precursor cells lack expression of the endogenous TCR/CD3 receptor and exhibit specific antitumor capacities. Oncoimmunology.

[90] Hübner J, Hoseini SS, Suerth JD, Hoffmann D, Maluski M, Herbst J (2016). Generation of Genetically Engineered Precursor T-Cells From Human Umbilical Cord Blood Using an Optimized Alpharetroviral Vector Platform. Molecular therapy : the journal of the American Society of Gene Therapy.

[91] Mohtashami M, Shah DK, Kianizad K, Awong G, Zúñiga-Pflücker JC (2013). Induction of T-cell development by Delta-like 4-expressing fibroblasts. Int Immunol.

[92] Shen S, Xu N, Symonds G, Dolnikov A (2016). Stem Cell Approach to Generate Cancer Specific Immune Effectors Cells. Int J Stem Cell Res Ther.

[93] Shen S, Xu N, Yang S, O'Brien T, Dolnikov A (2016). Stem Cell Approach to Generate Chimeric Antigen Receptor Modified Immune Effector Cells to Treat Cancer. Cytotherapy.

[94] Kwoczek J, Riese SB, Tischer S, Bak S, Lahrberg J, Oelke M (2017). Cord blood-derived T cells allow the generation of a more naïve tumor-reactive cytotoxic T-cell phenotype. Transfusion.

[95] Bunse M, Bendle GM, Linnemann C, Bies L, Schulz S, Schumacher TN (2014). RNAi-mediated TCR knockdown prevents autoimmunity in mice caused by mixed TCR dimers following TCR gene transfer. Molecular therapy : the journal of the American Society of Gene Therapy.

[96] ClinicalTrials.gov. A Study Evaluating UCART019 in Patients With Relapsed or Refractory CD19+ Leukemia and Lymphoma (Registry ID: NCT03166878): Chinese PLA General Hospital; 2017 [updated Jun 23, 2017. Available from: https://ClinicalTrials.gov/show/NCT03166878.

[97] ClinicalTrials.gov. Safety and Efficacy of ALLO-715 BCMA Allogenic CAR T Cells in in Adults With Relapsed or Refractory Multiple Myeloma (UNIVERSAL) (Registry ID: NCT04093596): Allogene Therapeutics; 2019 [updated Oct 14, 2021. Available from: https://ClinicalTrials.gov/show/NCT04093596.

[98] ClinicalTrials.gov. Safety and Efficacy of ALLO-501 Anti-CD19 Allogeneic CAR T Cells in Adults With Relapsed/Refractory Large B Cell or Follicular Lymphoma (ALPHA) (Registry ID: NCT03939026): Allogene Therapeutics; 2019 [updated Oct 14, 2021. Available from: https://ClinicalTrials.gov/show/NCT03939026.

[99] ClinicalTrials.gov. Anti-CD7 U-CAR-T Cell Therapy for T/NK Cell Hematologic Malignancies (Registry ID: NCT04264078) Xinqiao Hospital of Chongqing, Gracell Biotechnology Shanghai Co. Ltd. 2020 [updated Feb 11, 2020. Available from: https://ClinicalTrials.gov/show/NCT04264078.

[100] ClinicalTrials.gov. Safety and Efficacy of ALLO-501A Anti-CD19 Allogeneic CAR T Cells in Adults With Relapsed/Refractory Large B Cell Lymphoma (ALPHA-2) (Registry ID: NCT04416984): Allogene Therapeutics; 2020 [updated Oct 14, 2021. Available from: https://ClinicalTrials.gov/show/NCT04416984.

[101] Alarcón B, Gil D, Delgado P, Schamel WW (2003). Initiation of TCR signaling: regulation within CD3 dimers. Immunol Rev.

[102] Call ME, Wucherpfennig KW (2004). Molecular mechanisms for the assembly of the T cell receptor-CD3 complex. Mol Immunol.

[103] Chen S, Yang L, Lu X, Li B, Chan JY-H, Cai D (2010). Gene expression profiling of CD3γ, δ, ϵ, and ζ chains in CD4+ and CD8+ T cells from human umbilical cord blood. Hematol.

[104] Dietrich J, Neisig A, Hou X, Wegener AM, Gajhede M, Geisler C (1996). Role of CD3 gamma in T cell receptor assembly. J Cell Biol.

[105] Kuhns MS, Davis MM, Garcia KC (2006). Deconstructing the Form and Function of the TCR/CD3 Complex. Immunity.

[106] Schatz DG, Ji Y (2011). Recombination centres and the orchestration of V(D)J recombination. Nat Rev Immunol.

[107] Torikai H, Reik A, Liu PQ, Zhou Y, Zhang L, Maiti S (2012). A foundation for universal T-cell based immunotherapy: T cells engineered to express a CD19-specific chimeric-antigen-receptor and eliminate expression of endogenous TCR. Blood.

[108] ClinicalTrials.gov. Safety, Activity and Cell Kinetics of CYAD-211 in Patients With Relapsed or Refractory Multiple Myeloma (Registry ID: NCT04613557): Celyad Oncology SA; 2020 [updated Nov 30, 2020. Available from: https://ClinicalTrials.gov/show/NCT04613557.

[109] Anguille S, Al-Homsi AS, Deeren D, Nishihori T, Meuleman N, Abdul-Hay M, et al. Objective response at low dose in the first-in-human IMMUNICY-1 trial evaluating non-gene edited allogeneic CYAD-211 anti-BCMA CAR-T product in relapsed or refractory multiple myeloma. The Hague: EHA2021 Virtual: European Hematology Association; 2021.

[110] Osborn MJ, Webber BR, Knipping F, Lonetree CL, Tennis N, DeFeo AP (2016). Evaluation of TCR Gene Editing Achieved by TALENs, CRISPR/Cas9, and megaTAL Nucleases. Mol Ther.

[111] Bogdanove AJ, Voytas DF (2011). TAL Effectors: Customizable Proteins for DNA Targeting. Science.

[112] Joung JK, Sander JD (2013). TALENs: a widely applicable technology for targeted genome editing. Nat Rev Mol Cell Biol.

[113] Wah DA, Bitinaite J, Schildkraut I, Aggarwal AK (1998). Structure of FokI has implications for DNA cleavage. Proc Natl Acad Sci USA.

[114] Lamb BM, Mercer AC, Barbas CF (2013). Directed evolution of the TALE N-terminal domain for recognition of all 5' bases. Nucleic Acids Res.

[115] ClinicalTrials.gov. Study of UCART19 in Pediatric Patients With Relapsed/Refractory B Acute Lymphoblastic Leukemia (PALL) (Registry ID: NCT02808442): Institut de Recherches Internationales Servier; 2020 [updated Dec 24, 2020. Available from: https://ClinicalTrials.gov/show/NCT02808442.

[116] Qasim W, Zhan H, Samarasinghe S, Adams S, Amrolia P, Stafford S, Butler K, Rivat C, Wright G, Somana K, Ghorashian S, Pinner D, Ahsan G, Gilmour K, Lucchini G, Inglott S, Mifsud W, Chiesa R, Peggs KS, Chan L, Farzeneh F, Thrasher AJ, Vora A, Pule M, Veys P. Molecular remission of infant B-ALL after infusion of universal TALEN gene-edited CAR T cells. Sci Transl Med. 2017;9(374):eaaj2013. 10.1126/scitranslmed.aaj2013. 10.1126/scitranslmed.aaj2013 28123068

[117] Levitsky J, Leventhal JR, Miller J, Huang X, Chen L, Chandrasekaran D (2012). Favorable effects of alemtuzumab on allospecific regulatory T-cell generation. Hum Immunol.

[118] Cong L, Ran FA, Cox D, Lin S, Barretto R, Habib N (2013). Multiplex Genome Engineering Using CRISPR/Cas Systems. Science.

[119] Gasiunas G, Barrangou R, Horvath P, Siksnys V (2012). Cas9-crRNA ribonucleoprotein complex mediates specific DNA cleavage for adaptive immunity in bacteria. Proc Natl Acad Sci.

[120] Jinek M, Chylinski K, Fonfara I, Hauer M, Doudna JA, Charpentier E (2012). A Programmable Dual-RNA-Guided DNA Endonuclease in Adaptive Bacterial Immunity. Science.

[121] Fu Y, Foden JA, Khayter C, Maeder ML, Reyon D, Joung JK (2013). High-frequency off-target mutagenesis induced by CRISPR-Cas nucleases in human cells. Nat Biotechnol.

[122] Ihry RJ, Worringer KA, Salick MR, Frias E, Ho D, Theriault K (2018). p53 inhibits CRISPR-Cas9 engineering in human pluripotent stem cells. Nat Med.

[123] Amatya C, Pegues MA, Lam N, Vanasse D, Geldres C, Choi S (2021). Development of CAR T Cells Expressing a Suicide Gene Plus a Chimeric Antigen Receptor Targeting Signaling Lymphocytic-Activation Molecule F7. Mol Ther.

[124] Diaconu I, Ballard B, Zhang M, Chen Y, West J, Dotti G (2017). Inducible Caspase-9 Selectively Modulates the Toxicities of CD19-Specific Chimeric Antigen Receptor-Modified T Cells. Mol Ther.

[125] Duong MT, Collinson-Pautz MR, Morschl E, Lu A, Szymanski SP, Zhang M (2019). Two-Dimensional Regulation of CAR-T Cell Therapy with Orthogonal Switches. Mol Ther Oncol.

[126] Hoyos V, Savoldo B, Quintarelli C, Mahendravada A, Zhang M, Vera J (2010). Engineering CD19-specific T lymphocytes with interleukin-15 and a suicide gene to enhance their anti-lymphoma/leukemia effects and safety. Leukemia.

[127] Anzalone AV, Randolph PB, Davis JR, Sousa AA, Koblan LW, Levy JM (2019). Search-and-replace genome editing without double-strand breaks or donor DNA. Nature.

[128] Gaudelli NM, Komor AC, Rees HA, Packer MS, Badran AH, Bryson DI (2017). Programmable base editing of A•T to G•C in genomic DNA without DNA cleavage. Nature.

[129] Komor AC, Kim YB, Packer MS, Zuris JA, Liu DR (2016). Programmable editing of a target base in genomic DNA without double-stranded DNA cleavage. Nature.

[130] Nianias A, Themeli M (2019). Induced Pluripotent Stem Cell (iPSC)-Derived Lymphocytes for Adoptive Cell Immunotherapy: Recent Advances and Challenges. Curr Hematol Malig Rep.

[131] Pan J, Tan Y, Wang G, Deng B, Ling Z, Song W, et al. Donor-Derived CD7 Chimeric Antigen Receptor T Cells for T-Cell Acute Lymphoblastic Leukemia: First-in-Human, Phase I Trial. J Clin Oncol. 2021;39(30):3340–51. 10.1200/JCO.21.00389 34324392

[132] Wang X, Li S, Gao L, Yuan Z, Wu K, Liu L (2020). Abstract CT052: Clinical safety and efficacy study of TruUCAR™ GC027: The first-in-human, universal CAR-T therapy for adult relapsed/refractory T-cell acute lymphoblastic leukemia (r/r T-ALL). Cancer Res.

[133] Li S, Gao L, Yuan Z, Wu K, Liu L, Luo L (2021). Abstract LB147: Updates on clinical safety and efficacy result of GC027, the first-in-human, "Off-the-Shelf" CD7 CAR-T stand-alone therapy for adult patients with relapsed/refractory T-cell lymphoblastic leukemia (r/r T-ALL). Cancer Res.

[134] Georgiadis C, Rasaiyaah J, Gkazi SA, Preece R, Etuk A, Christi A, Qasim W. Base-edited CAR T cells for combinational therapy against T cell malignancies. Leukemia. 2021;35(12):3466–81. 10.1038/s41375-021-01282-6. 10.1038/s41375-021-01282-6 PMC8632682 34035409

[135] ClinicalTrials.gov. NY-ESO-1-redirected CRISPR (TCRendo and PD1) Edited T Cells (NYCE T Cells) (Registry ID: NCT03399448): University of Pennsylvania, Parker Institute for Cancer Immunotherapy, Tmunity Therapeutics; 2018 [updated Oct 12, 2020. Available from: https://ClinicalTrials.gov/show/NCT03399448.

[136] Stadtmauer EA, Fraietta JA, Davis MM, Cohen AD, Weber KL, Lancaster E, Mangan PA, Kulikovskaya I, Gupta M, Chen F, Tian L, Gonzalez VE, Xu J, Jung IY, Melenhorst JJ, Plesa G, Shea J, Matlawski T, Cervini A, Gaymon AL, Desjardins S, Lamontagne A, Salas-Mckee J, Fesnak A, Siegel DL, Levine BL, Jadlowsky JK, Young RM, Chew A, Hwang WT, Hexner EO, Carreno BM, Nobles CL, Bushman FD, Parker KR, Qi Y, Satpathy AT, Chang HY, Zhao Y, Lacey SF, June CH. CRISPR-engineered T cells in patients with refractory cancer. Science. 2020;367(6481):eaba7365. 10.1126/science.aba7365. 10.1126/science.aba7365 PMC11249135 32029687

[137] Lanza F, Maffini E, Rondoni M, Massari E, Faini AC, Malavasi F (2020). CD22 Expression in B-Cell Acute Lymphoblastic Leukemia: Biological Significance and Implications for Inotuzumab Therapy in Adults. Cancers.

[138] Hu Y, Zhou Y, Zhang M, Ge W, Li Y, Yang L (2021). CRISPR/Cas9-Engineered Universal CD19/CD22 Dual-Targeted CAR-T Cell Therapy for Relapsed/Refractory B-cell Acute Lymphoblastic Leukemia. Clin Cancer Res.

[139] Eyquem J, Mansilla-Soto J, Giavridis T, van der Stegen SJ, Hamieh M, Cunanan KM (2017). Targeting a CAR to the TRAC locus with CRISPR/Cas9 enhances tumour rejection. Nature.

[140] Kagoya Y, Guo T, Yeung B, Saso K, Anczurowski M, Wang CH, Murata K, Sugata K, Saijo H, Matsunaga Y, Ohashi Y, Butler MO, Hirano N. Genetic Ablation of HLA Class I, Class II, and the T-cell Receptor Enables Allogeneic T Cells to Be Used for Adoptive T-cell Therapy. Cancer Immunol Res. 2020;8(7):926–36. 10.1158/2326-6066.CIR-18-0508. 10.1158/2326-6066.CIR-18-0508 32321775

[141] Kamiya T, Wong D, Png YT, Campana D (2018). A novel method to generate T-cell receptor-deficient chimeric antigen receptor T cells. Blood Adv.

[142] Stenger D, Stief TA, Käuferle T, Willier S, Rataj F, Schober K, et al. Endogenous TCR promotes in vivo persistence of CD19-CAR-T cells compared to a CRISPR/Cas9-mediated TCR knockout CAR. Blood. 2020. 10.1182/blood.2020005185 PMC7612202 32483603

[143] Roth TL, Puig-Saus C, Yu R, Shifrut E, Carnevale J, Li PJ (2018). Reprogramming human T cell function and specificity with non-viral genome targeting. Nature.

[144] ClinicalTrials.gov. Safety and Efficacy of ALLO-605 an Anti-BCMA Allogeneic CAR T Cell Therapy in Patients With Relapsed/Refractory Multiple Myeloma (IGNITE) (Registry ID: NCT05000450): Allogene Therapeutics; 2021 [updated Oct 14, 2021. Available from: https://ClinicalTrials.gov/show/NCT05000450.

[145] ClinicalTrials.gov. Safety and Efficacy of ALLO-316 in Subjects With Advanced or Metastatic Clear Cell Renal Cell Carcinoma (Registry ID: NCT04696731) (TRAVERSE): Allogene Therapeutics; 2021 [updated Oct 14, 2021. Available from: https://ClinicalTrials.gov/show/NCT04696731.

[146] Allogene. [Press Release] Allogene Therapeutics Reports FDA Clinical Hold of AlloCAR T Trials Based on a Single Patient Case in ALPHA2 Trial: Allogene Therapeutics; 2021 [updated Oct 7, 2021. Available from: https://ir.allogene.com/news-releases/news-release-details/allogene-therapeutics-reports-fda-clinical-hold-allocar-t-trials.

[147] Allogene. [Press Release] Allogene Therapeutics Reports Third Quarter 2021 Financial Results and Business Update: Allogene Therapeutics; 2021 [updated Nov 4, 2021. Available from: https://ir.allogene.com/news-releases/news-release-details/allogene-therapeutics-reports-third-quarter-2021-financial.

[148] Allogene. [Press Release] Allogene Therapeutics Announces Removal of FDA Clinical Hold Across All AlloCAR T™ Clinical Trials: Allogene Therapeutics; 2021 [updated Jan 10, 2022. Available from: https://ir.allogene.com/news-releases/news-release-details/allogene-therapeutics-announces-removal-fda-clinical-hold-across.

